# An outbreak of post-acupuncture cutaneous infection due to *Mycobacterium abscessus*

**DOI:** 10.1186/1471-2334-6-6

**Published:** 2006-01-13

**Authors:** Joon Young Song, Jang Wook Sohn, Hye Won Jeong, Hee Jin Cheong, Woo Joo Kim, Min Ja Kim

**Affiliations:** 1 Division of Infectious Disease, Department of Internal Medicine, Korea University College of Medicine, Seoul, Republic of Korea

## Abstract

**Background:**

Despite the increasing popularity of acupuncture, the importance of infection control is not adequately emphasized in Oriental medicine. In December 2001, an Oriental medical doctor in Seoul, South Korea, encountered several patients with persistent, culture-negative skin lesions on the trunk and extremities at the sites of prior acupuncture treatment. We identified and investigated an outbreak of *Mycobacterium abscessus *cutaneous infection among the patients who attended this Oriental medicine clinic.

**Methods:**

Patients were defined as clinic patients with persistent cutaneous infections at the acupuncture sites. Medical records for the previous 7 months were reviewed. Clinical specimens were obtained from the patients and an environmental investigation was performed. *M. abscessus *isolates, cultured from patients, were compared by pulsed-field gel electrophoresis (PFGE).

**Results:**

Forty patients who attended the Oriental medicine clinic and experienced persistent cutaneous wound infections were identified. Cultures from five of these patients proved positive, and all other diagnoses were based on clinical and histopathologic examinations. All environmental objects tested were negative for *M. abscessus*, however, most were contaminated by various nosocomial pathogens. Molecular analysis using PFGE found all wound isolates to be identical.

**Conclusion:**

We have identified a large outbreak of rapidly growing mycobacterial infection among patients who received acupuncture at a single Oriental medicine clinic. Physicians should suspect mycobacterial infections in patients with persistent cutaneous infections following acupuncture, and infection control education including hygienic practice, should be emphasized for Oriental medical doctors practicing acupuncture.

## Background

Acupuncture has become increasingly accepted as a form of alternative medicine in Western countries, though it has been practiced for hundreds of years by Oriental medical doctors in South Korea. As of 2002, about 13,000 Oriental medical doctors were licensed through the national examination for medical practice. These practitioners are recognized as a type of medical doctor by the South Korean public, however, Oriental medical doctors and other medical doctors are educated, and subsequently practice, in a separate system. Therefore, Oriental medical doctors are relatively unfamiliar with infection control, and usually see patients in general office rooms.

Acupuncture can be complicated by infections caused by environmental microorganisms or patient skin flora [1-3]. Although there have been several acupuncture-associated hepatitis B outbreaks [4,5], no acupuncture related outbreak of bacterial infections has been reported.

*Mycobacterium abscessus *is a rapidly growing mycobacterium, which is widespread in natural and drinking water [6,7]. These species are relatively resistant to chlorine and glutaraldehyde [7-10], thus, are frequently detected in hospital tap water [6-8,11,12].

These organisms most commonly cause soft-tissue infection (community acquired or nosocomial) following minor trauma or intramuscular injection. In addition, these organisms have been implicated in surgical wound infections, bacteremia associated with dialysis, catheter-related sepsis and chronic ear infections [8,12,13].

There have been sporadic cases reported over the past few years in which non-tuberculous mycobacterial infections complicate acupuncture [9,14,15]. Herein, we report on an outbreak of post-acupuncture cutaneous infection, due to *M. abscessus*, in an Oriental clinic.

## Methods

### Description of the outbreak

From August to November 2001, an Oriental medical doctor in a one-doctor office in Seoul, South Korea observed nodular lesions and abscesses at acupuncture sites in several patients. Patients were referred to a local clinic where, initially, the lesions were treated with antibiotics and abscesses were drained, however, no improvement was observed. At the end of December 2001, patients were subsequently referred to the infectious disease section of Korea University Hospital, where the acupuncture associated abscesses were diagnosed as *M. abscessus *infection.

Medical records were reviewed, and all patients who received acupuncture from June to December 2001 were informed. A case was defined as any person with erythematous skin papules, nodules or abscesses in the acupuncture area, who received acupuncture at a specific Oriental medicine clinic between June and December 2001. A careful clinical history and physical examination were taken, and skin lesion cultures and biopsies were performed on as many patients as possible. Furthermore, details of the acupuncture procedure and clinical environment information were gathered from both the Oriental medical doctor and patients.

### Environmental investigation

Thirty-five environmental samples were collected from the Oriental medicine clinic, which included the hands and anterior nares of healthcare workers and numerous substances that had probably come in contact with the skin lesions of the patients, such as: disposable needles, hot packs, towels for hot packs, alcohol-retained bottles, ultrasound gel, and boiling tank water. Culture samples were also obtained from anything that may have come in contact with the patient skin lesions, such as: the basins, bottle inlets, and other objects. These samples were tested for the presence of mycobacterial cultures. The clinic office area did not have any plants or animals.

### Laboratory tests

Clinical specimens were obtained from patients by needle aspiration or biopsy, depending on the character of the lesion. In addition, multiple environmental specimens from the Oriental medicine clinic were obtained as described above. Samples were plated on Ogawa and blood agar and incubated for 7 days at 30°C.

When yellow-colored colonies formed on Ogawa medium in the clinical specimens, culture samples were sent to the National Tuberculosis Institute for species isolation. Environmental specimen isolation was performed at the Korea University Anam Hospital microbiology laboratory. The biopsy specimens were examined histopathologically.

Mycobacterial species were identified and differentiated by colony morphology, biochemical tests and molecular biological methods, including: catalase test, nitrate reduction test, sodium chloride tolerance test, and polymerase chain reaction amplification-restriction analysis (PRA) of *rpoB *[16].

### Molecular comparison

Pulsed-field gel electrophoresis (PFGE) of large genomic DNA restriction (*Xba*I) fragments was performed as described previously [17,18], and results were interpreted based on the criteria set forth by Tenover et al [19].

## Results

### Description of the outbreak

The index case presented with a skin lesion in early September 2001, a few weeks after acupuncture treatment. Among the patients who received acupuncture between August and November 2001, some subsequently experienced skin lesions or abscess at the acupuncture site. A total of forty cases were identified.

The outbreak involved a single Oriental medical doctor and three assistant nurses. The medical practice included acupuncture usually followed by hot pack and ultrasound therapy. The hot pack was applied over the towels, and ultrasound therapy was carried out after spreading gel over the skin. Acupuncture was performed with disposable needles which were not reused.

**Figure 1:**
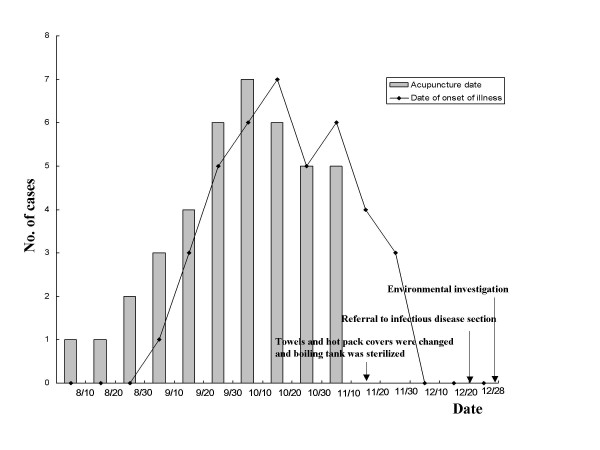
**Cases of post-acupuncture *Mycobacterium abscessus *cutaneous infection in an Oriental medicine clinic during an outbreak between September and November 2001**. The patients started to receive acupuncture in early August, and the index case was identified in early September. Most cases were concentrated between September and October. After sterilization and a regular towel change in mid-November, no further cases occurred.

In mid-November, before an investigation of the outbreak, the Oriental medical doctor changed the towels and hot pack covers to new towels and covers and sterilized the boiling tank (figure 1). The outbreak promptly ceased.

**Figure 2:**
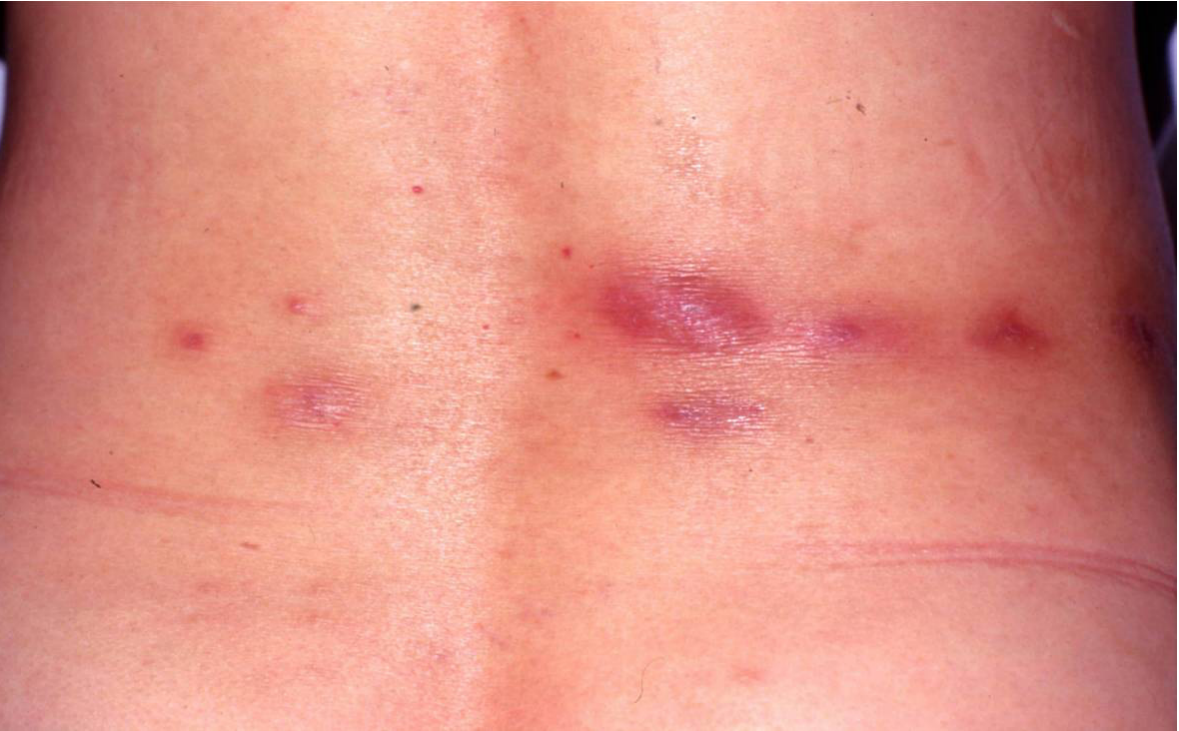
The back of a 58-year-old woman with typical disease presentation.

The majority of patients were female (33 females, 7 males), with a median age of 43 years (range, 23–61 years). The incubation period varied from 3 to 60 days (mean, 16 days). The average time to lesion diagnosis at the Korea University Hospital was 132 days (range, 58–257 days). All 40 patients complained of a burning sensation around the skin lesion, and 16 patients also experienced an itching sensation. Systemic symptoms such as fever or a chill were absent from all patients. Various skin manifestations were observed. Erythematous papular, nodular skin lesions were noted in some patients, while ulcerative, abscess-forming lesions were seen in others (figure 2). The wound distribution was as follows: back (27 patients, 67.5%), lower extremity (13 patients, 32.5%), shoulder (5 patients, 12.5%), buttocks (5 patients, 12.5%), neck (2 patients, 5%), axilla (1 patient, 2.5%) and inguinal area (1 patient, 2.5%). Nine patients had skin lesions at two or more sites.

Culture specimens were obtained by needle aspiration in 8 of 40 patients. The bacterium was recovered on Ogawa medium from 5 (63%) of the 8 samples. All of these isolates were submitted to the National Tuberculosis Institute, where they were identified as *M. abscessus *using standard biochemical methods and *rpoB *PRA. The remaining 35 patients were diagnosed based on clinical and histopathologic examination of the lesions. Biopsy specimens showed mixed suppurative and granulomatous inflammation with foreign bodies. Caseous necrosis was not observed.

Thirty-eight of 40 patients received oral clarithromycin (500 mg, twice daily) for 3–6 months, which resolved the skin wounds without complications. Amikacin (three times per week) was administered concurrently in 25 patients. One patient received rifampin in combination with oral clarithromycin. The lesions were excised in two patients and no antibiotics were given. Hospitalization was not necessary, and patients treated with clarithromycin did not receive any surgical management.

### Environmental factors

The environmental samples were obtained at the end of December 2001 (figure 1). The samples may not accurately represent the environmental conditions at the time of infection because medical devices were sterilized and towels were changed in mid-November, before the investigation. Environmental cultures obtained from the healthcare workers, disposable needles, towels, hot packs, alcohol-retained bottles, ultrasound gel, boiling tank water, and several other environmental objects were negative for *M. abscessus*, although most cultures were contaminated by various nosocomial pathogens, such as: *Pseudomonas aeruginosa*, *Corynebacterium *spp. and *Bacillus subtilis*.

### Molecular comparison

**Figure 3:**
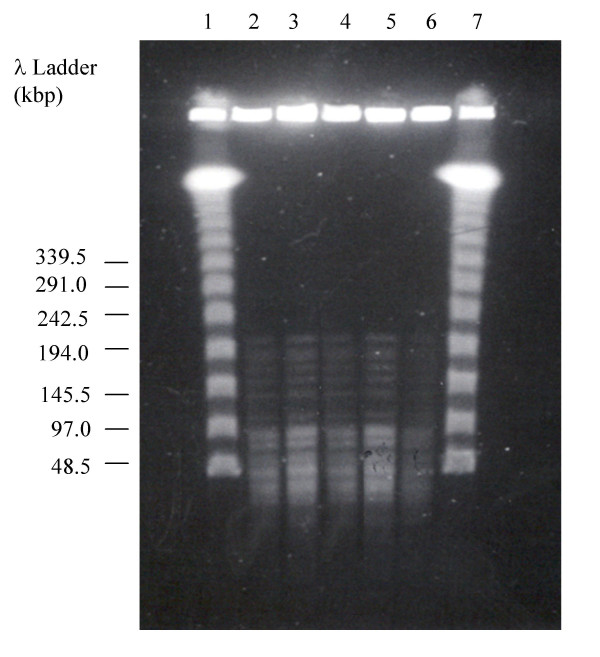
**Pulsed-field gel electrophoresis of representative isolates from patients, digested with the restriction enzyme *Xba*I**. Lanes 2 – 6 show *M. abscessus *isolates from five patients, lanes 1 and 7 are a 48.5-kb molecular-weight marker.

*M. abscessus *isolates from five patients' skin wounds were compared using PFGE. The five isolates were indistinguishable (representative isolates are shown in figure 3).

## Discussion

We reported an outbreak of acupuncture-associated abscesses due to rapidly growing mycobacteria. Outbreaks of injection-related mycobacteria have been described following injections of histamine, lidocaine, saline solution, vaccines, disinfectant solution and adrenal cortex extract [20-22]. The main distinguishing feature of this study is that no material was injected with the acupuncture needle.

The exact source of *M*. *abscessus *in this outbreak was not determined, however, there are several possible explanations. First, the acupuncture needle itself could be the source of infection, likely due to needle length. Acupuncture consists of solid needles, ranging from 15 to 50 mm long, piercing the skin [23]. The acupuncture-associated mycobacteriosis occured most often on the back and the knee-joint areas, where the needles are deeply inserted. Although many patients received acupuncture at the ankle and wrist areas, none of these patients developed a skin lesion. A second possibility is skin contamination before or after acupuncture. In general, the Oriental medical doctor rubbed alcohol on the patient's skin to sterilize it before acupuncture. Patients were also exposed to hot-pack therapy and gel (mixture of Antiphramin^® ^and ultrasound jelly) massage after acupuncture. Therefore, cultures from environmental objects, including: disposable needles, hot packs, towels, Antiphramin^® ^gel, alcohol-retained bottles and boiling tank water, were obtained. Although these samples gave negative cultures, towels and hot-pack covers are still a possible main source of infection. Mycobacteria may have been introduced to soft tissue by contaminated towels or hot-pack covers via the acupuncture site. No further cases have been seen since mid-November, after sterilization and regular towel change (figure 1). Finally, though unlikely, the needle could have been carelessly exposed to tap water.

Since the symptoms are relatively mild and indolent, the clinical diagnosis of mycobacteriosis is often delayed. In contrast to other pyogenic bacterial infections with a shorter incubation period, infections due to rapidly growing mycobacteria have longer incubation periods (several days to several months) [12,14,21,22]. It took more than 2 months from initial manifestation to diagnosis, which was also observed in previous study [21]. This suggests that careful monitoring is required for early diagnosis and appropriate treatment. Thirty-eight of 40 patients were treated successfully with administration of oral clarithromycin (500 mg, twice daily) for 3–6 months and some of these patients also received either amikacin or rifampin. None of the patients required hospitalization or surgical management. The mean duration of antibiotic treatment varied from 9 to 12 months in several previous studies, however, other studies saw complete healing after 2–3 months of antibiotic treatment with incision and drainage [15,21]. This indicates that several factors affect the prognosis, such as the disease type (e.g., cellulite vs. abscess) and the underlying disease. Therefore standard guidelines including antibiotic treatment and surgical management should be established.

With the increasing popularity of acupuncture, a number of surveys concerning the safety of acupuncture have been conducted. Various adverse events were reported, such as: needle pain, tiredness, bleeding, headache, faintness and rare pneumothorax [24,25]. The importance of infection control has not been emphasized in Oriental medicine despite several sporadic reports of secondary wound infection at acupuncture sites. Cho et al. reported a case of a serious retroperitoneal abscess following acupuncture [26]. Rapidly growing mycobacteria are well known to be resistant to chlorhexidine and relatively resistant to alcohol [27]. The outbreak of infection has been attributed to improper sterilization rather than to iodine and alcohol resistance [20,27].

There are several limitations that prevented us from identifying the exact contamination source. Because the hot-pack boiling tank was sterilized and the towels and hot-pack covers were changed a few weeks prior to this study, the environmental source of the rapidly growing mycobacteria and their mechanism of distribution were not elucidated by this study. We could not compare patients with controls. Because the Oriental medicine clinic was an outpatient setting and the medical records were poor there was no efficient way to assess unaffected persons.

In conclusion, it is important to educate Oriental medical doctors to wash their hands properly with alcoholic chlorhexadin or povidone iodine, to clean the proposed skin site with povidone iodine or non-contaminated alcohol and allow sufficient drying time, and to sterilize both the environment and equipment. We recommend that Oriental medicine courses provide infection control education. Moreover, future epidemiologic studies are needed to elucidate the exact mechanism of infection.

## Conclusion

To our knowledge, this is the first outbreak of acupuncture-associated wound infection caused by rapidly growing mycobacteria. Physicians should suspect mycobacterial infections in patients with persistent cutaneous infections following acupuncture. Furthermore, education on infection control, including hygienic practice, should be emphasized to Oriental medical doctors practicing acupuncture.

## Competing interests

The author(s) declare that they have no competing interests.

## Authors' contributions

JYS participated in the study design and composed the manuscript. JWS planned and coordinated the study and helped draft the manuscript. HWJ participated in the epidemiologic surveillance and molecular studies. HJC and WJK participated in the study design and epidemiologic surveillance. MJK participated in the study design and performed the molecular studies. All authors read and approved the final manuscript.

## Pre-publication history

The pre-publication history for this paper can be accessed here:


